# First record of a by-the-wind-sailor (*Velella velella* Linnaeus, 1758) in the Galápagos Archipelago - Ecuador

**DOI:** 10.3897/BDJ.7.e35303

**Published:** 2019-08-21

**Authors:** Martín Carrera, José E Trujillo, Margarita Brandt

**Affiliations:** 1 Granados 374 y Eloy Alfaro, Conjunto Camino Real, Quito, Ecuador Granados 374 y Eloy Alfaro, Conjunto Camino Real Quito Ecuador; 2 Puerto Azul Mz C7 V14, 090112 , Guayaquil, Ecuador Puerto Azul Mz C7 V14, 090112 Guayaquil Ecuador; 3 Universidad San Francisco de Quito/Galápagos Science Center, Quito, Ecuador Universidad San Francisco de Quito/Galápagos Science Center Quito Ecuador

**Keywords:** *Velella velella*, Galapagos Archipelago, first record

## Abstract

We present the first official record of the by-the-wind-sailor (*Velella velella*) for Ecuador. Twelve individuals were found along different beaches of San Cristóbal and Santa Cruz Islands in Galápagos Archipelago, Ecuador. These sightings may be influenced by El Niño Southern Oscillation events.

## Introduction

*Velella velella* (Linnaeus, 1758) is a holoplanktonic athecate hydroid (Hydrozoa: Anthoathecata) from the Porpitidae family that is well known as “by-the-wind-sailor” (World Register of Marine Species 2018; World Hydrozoa Database 2019) due to its easily recognisable sail, which helps individuals to disperse over the ocean surface via wind currents (Purcell et al. 2012). *Velella velella* floats on the ocean surface during the asexual colonial stage where it primarily feeds on copepods and small fishes. In addition, it harbours symbiotic zooxanthellae that provide extra nutrition to the host (Purcell et al. 2012). It is also known by their occasional mass strandings in beaches, where millions of individuals become a great source of organic material to the shoreline (Kemp 1986; Flux 2008).

Even though *V. velella* could potentially have a cosmopolitan distribution due to its sail (i.e. aid for dispersal), its common distribution is in the northern hemisphere in the Pacific and Atlantic Oceans, as well as in the Mediterranean Sea (World Register of Marine Species 2018). For the Pacific Ocean, there are several informal records (i.e. iNaturalist records) in Canada, Australia and Mexico (Baldwin 2017; Beuzeville 2018; Navarro 2018) and two formal records for New Zealand (Flux 2008) and the United States of America (Zeman et al. 2018). For the Pacific Coast of South America, the only two records of *V. velella* come from Chile (Moyano and Valdovinos 1984; Araya and Aliaga 2018). Checklists on cnidarians, including hydromedusae, from other countries of South America do not mention its occurrence (e.g. from Colombia: Baldrich and López 2010; Baldrich and López 2013; Smithsonian Institute 2018, from Ecuador: Andrade 2010; Andrade 2012; Chiriboga et al. 2016). Here, we report two separate sightings of the "by-the-wind sailor" in the Galápagos Islands, being the first record for Ecuador.

## Results and discussion

During 2017 and 2018, separate sightings of *Velella velella* were recorded in two islands of the Galápagos Archipelago, off the coast of Ecuador (approx. 960 km to the west of South America). On 28 August 2017, approximately eight individuals of *V. velella* were spotted on La Lobería beach (0°55'36.64''S; 89°36'41.88''W) at San Cristóbal Island. We photographed one individual that corresponded to a “right-by-the-wind-sailor” due to the direction of its sail (Fig. 1). Almost a year later, on 16 June 2018, several individuals of *V. velella* were observed washed ashore on Tortuga Bay (0°45'40.5''S; 90°20'05.5''W), a beach at Santa Cruz Island. Although no exact counts are available, at least four different individuals were photographed (Fig. 2). Most individuals from Santa Cruz were right-by-the-wind-sailors. However, this was difficult to assess in one individual whose sail was not developed (individual "two", Fig. 2 c). All individuals from both San Cristóbal and Santa Cruz Islands were of small size, ranging from a few mm to no more than 2 cm.

Southwest orientated, Tortuga Bay is a dissipative beach with a gentle slope exposed to southern swells. These features make Tortuga Bay a trap for drifting organisms when winds hit from the south. In this respect, we also observed several specimens of *Porpita porpita* (Linnaeus, 1758) and *Physalia physalis* (Linnaeus, 1758) washed up along with *V. velella* (Fig. 3). These multispecies strandings seem to be common (e.g. Thiel and Gutow 2005; Flux 2008).

To the best of our knowledge, this is the first official record of *V. vellella* in Ecuador. We suggest that they are likely uncommon in the Galápagos Archipelago. Araya and Aliaga (2018) reported that El Niño Southern Oscillation (ENSO) events are strongly correlated with blooms of jellyfishes and related fauna in the south-eastern coast of South America. In 2017 and 2018, a mild ENSO was detected in the region (ENFEN 2017; ENFEN 2018; World Meteorological Organization 2018). The changes in the intensities of trade winds and, hence, in the weather, was not extreme but these anomalies could explain why *V. velella* arrived at the Galápagos (ENFEN 2017; Araya and Aliaga 2018).

## Figures and Tables

**Figure 1a.**
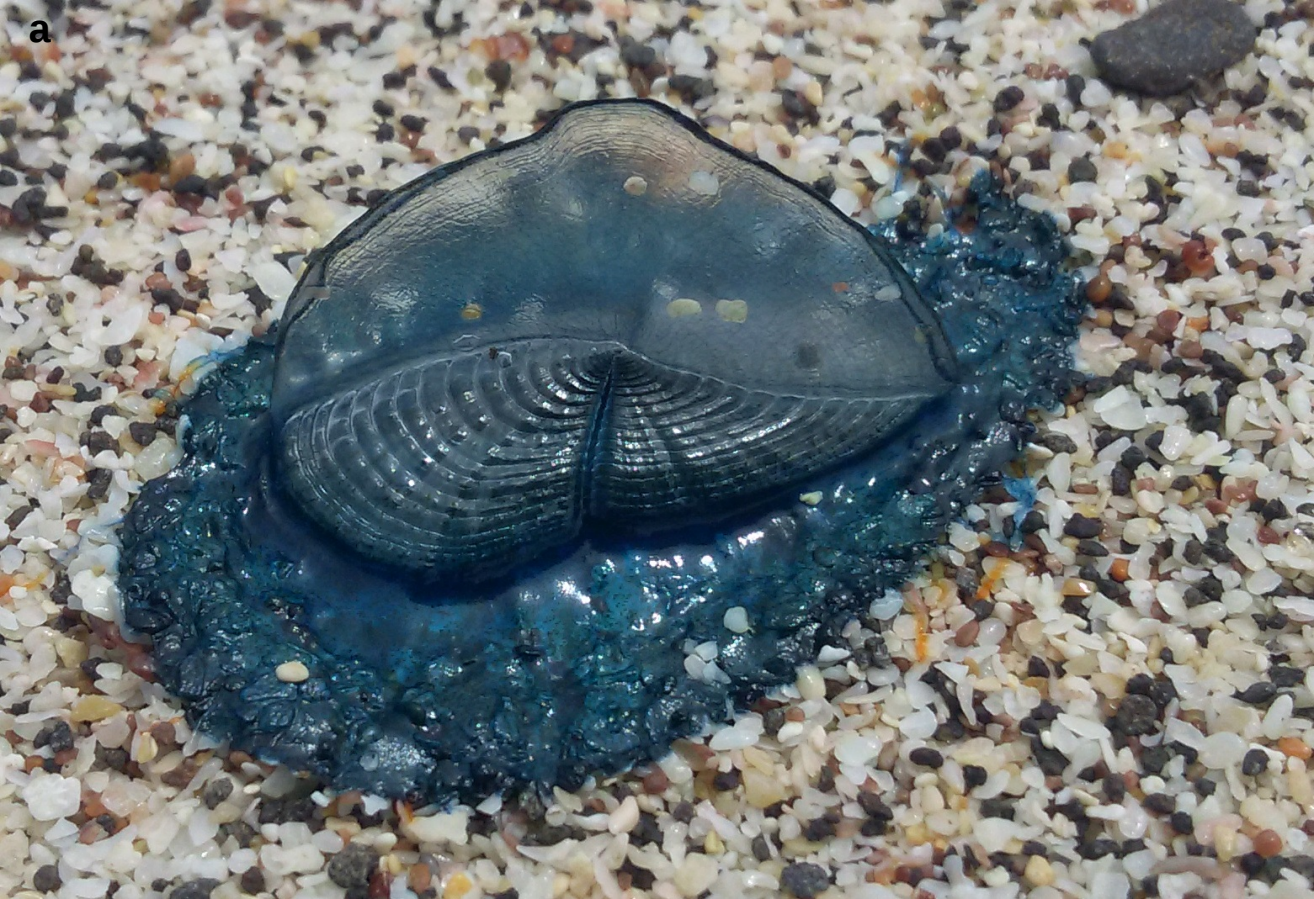
side view

**Figure 1b.**
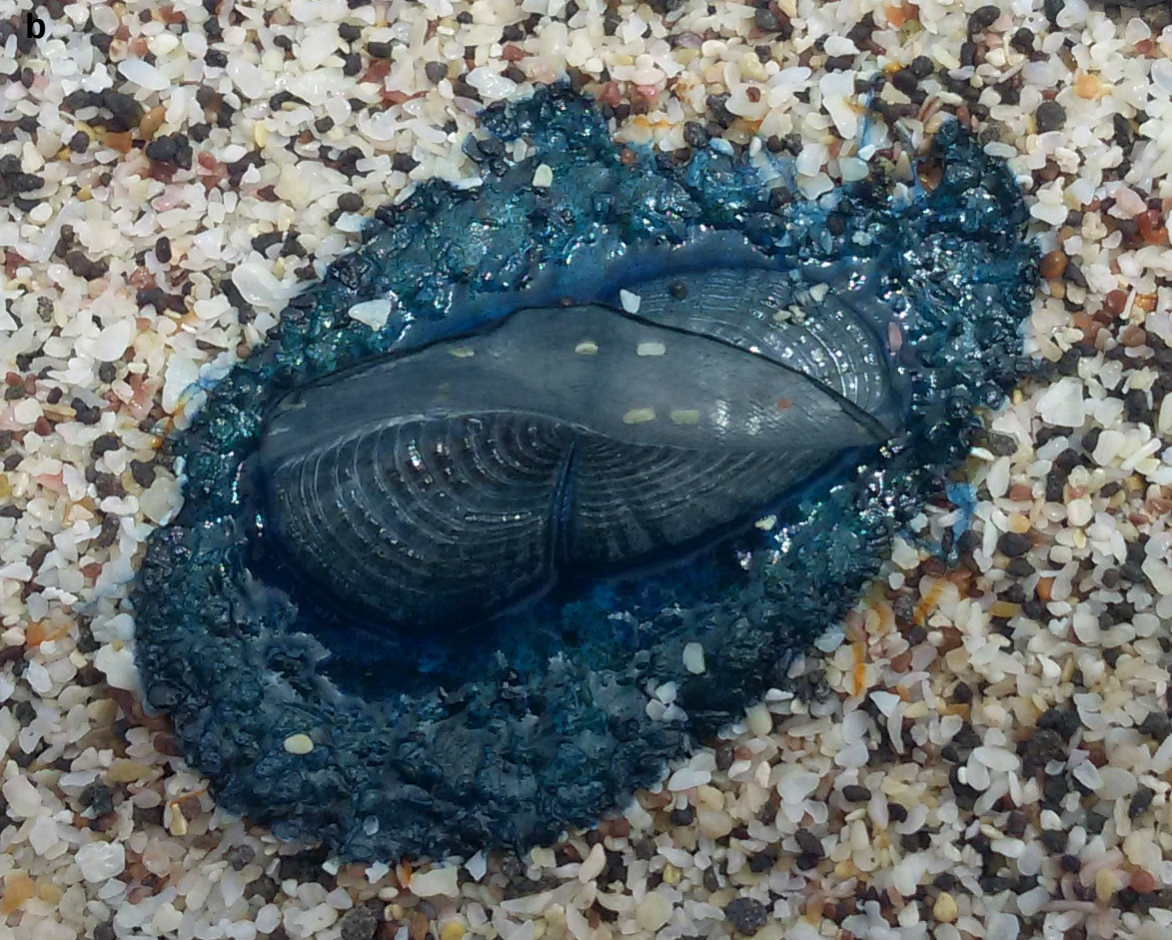
dorsal view with the right-sided sail

**Figure 2a.**
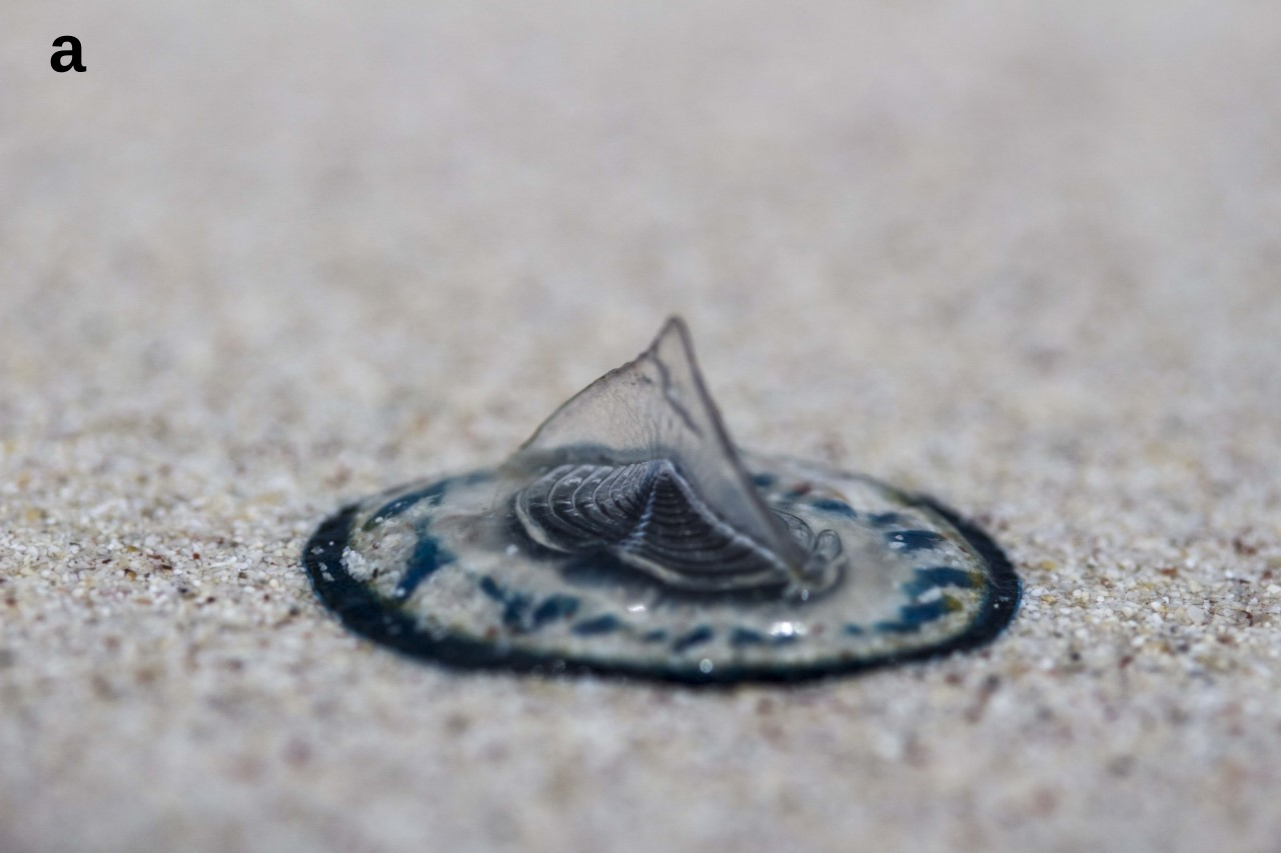
*V. velella*, individual "one"

**Figure 2b.**
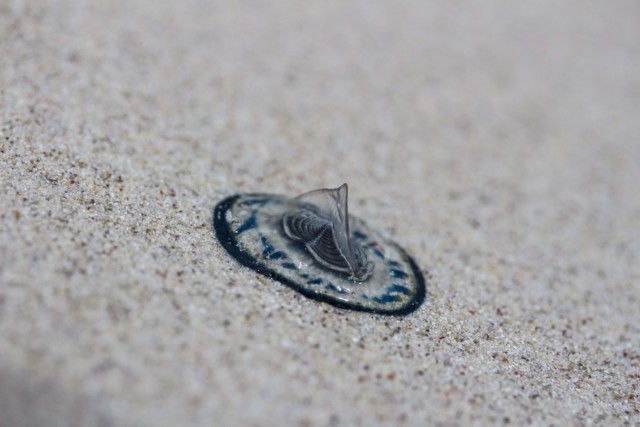
*V. velella*, individual "one"

**Figure 2c.**
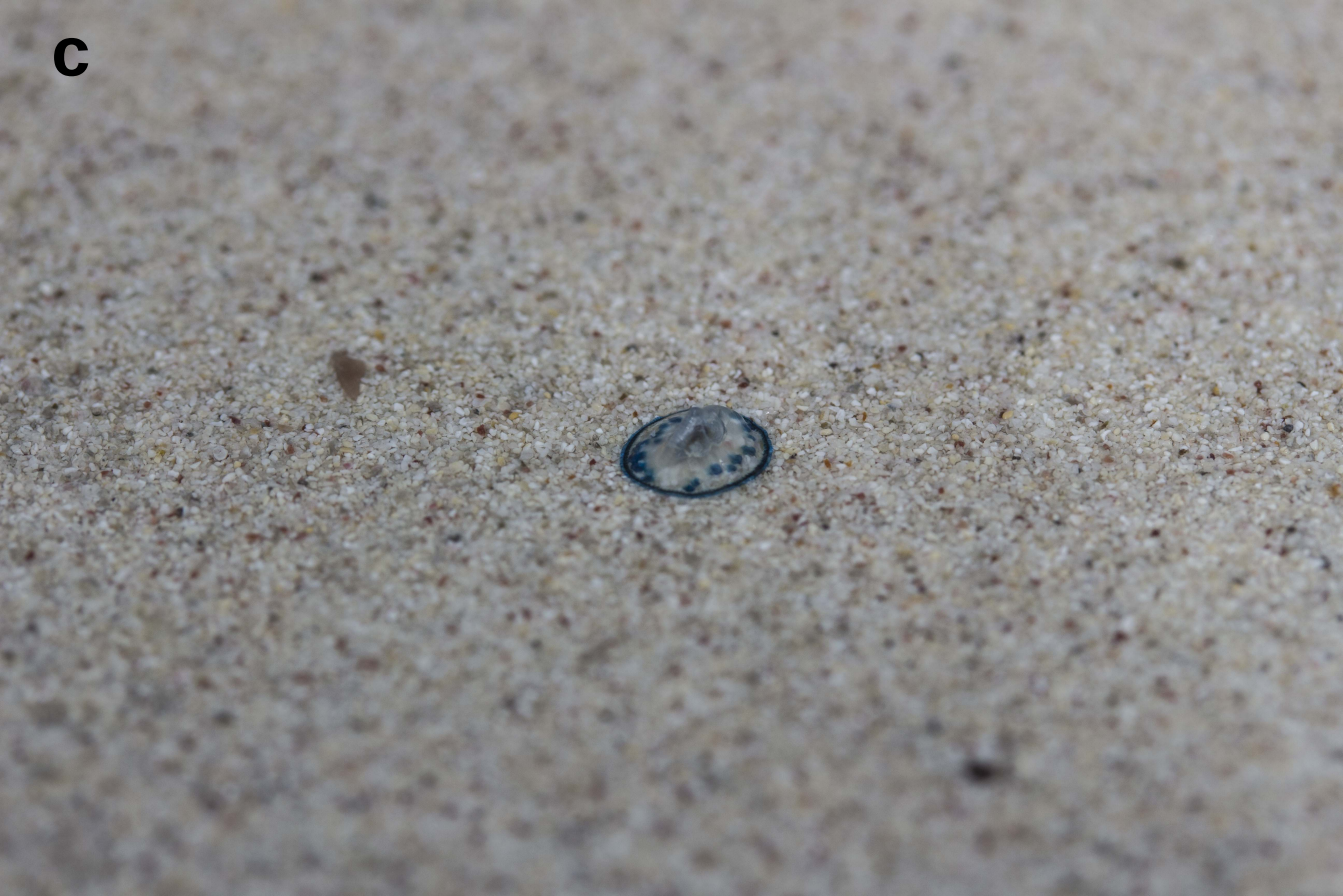
*V. velella*, individual "two"

**Figure 2d.**
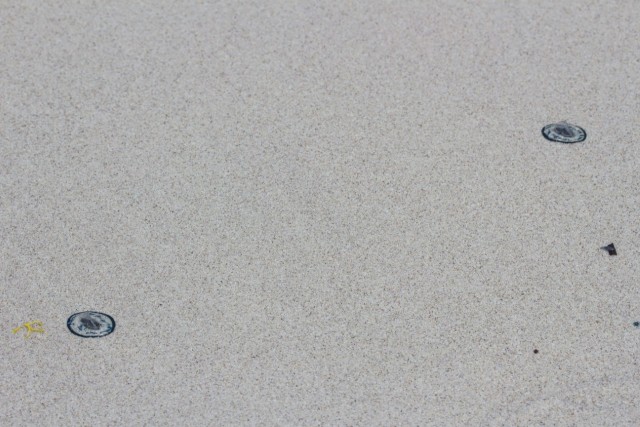
*V. velella*, individuals "three" and "four"

**Figure 3a.**
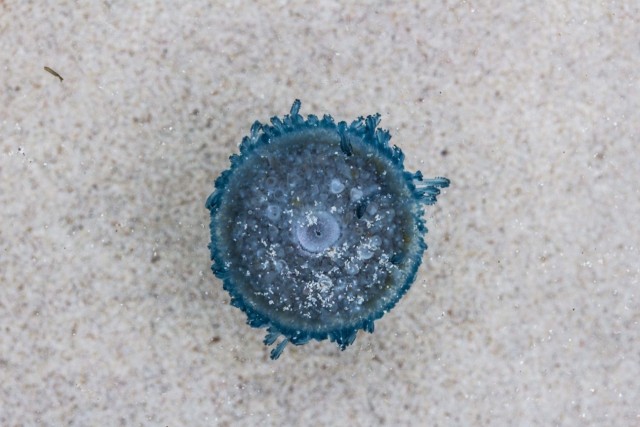
*Porpita porpita*, ventral view

**Figure 3b.**
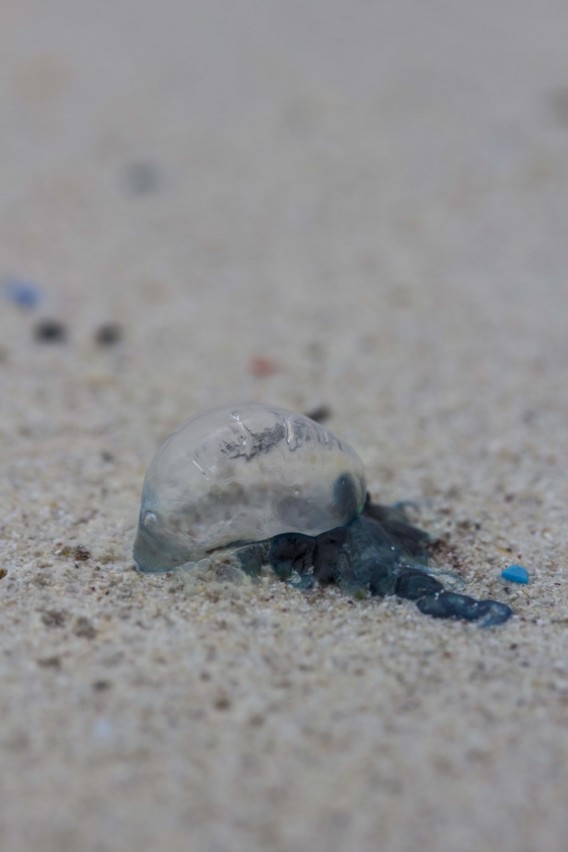
*Physalis physalis*, side view
